# A Case of Rheumatic Carditis Suddenly Fatal

**Published:** 1873-08-01

**Authors:** 


### A CASE OF RHEUMATIC CARDITIS SUDDENLY FATAL.

BY H. A. JOHNSON, M.D., AND PROFESSOR, CHICAGO, ILLINOIS.

I saw Mrs. S., age 31, first on January 9th, 1873, early in the afternoon. I found her suffering from ill-defined muscular soreness, especially over the abdomen and in the back. Bowels not disturbed; skin moist; pulse slightly accelerated; tongue moist and white. I prescribed the following:

℞.—Potass. bromid., ʒ iij.

Aquæ camph., fl. ℥ iv.

M. sig. One tablespoonful every three hours.

Jan. 10th—Saw her in the afternoon. She passed a restless night; some nausea this morning, which she attributes to the medicine; supposed that she had taken morphine or opium. Pain more decidedly rheumatic; hips and knees sore; motion difficult; menses came on during the night, or this morning—several days earlier than expected; begs for something to allay pain, which was now almost unbearable. I directed the following:

℞.—Hydr. sub. mur., grs. iv.

Morph. sulph., gr. i.

Sodæ bi carb., ℈i.

M. Ft. pulv. no. vi.

S. One every three hours.

January 11th, P. M.—Pain still continues in hips and knees; she attributes a part of it to the advent of the menses; condition of the stomach improved. Has rested and slept some under the influence of the powders of morphine, sub muriate and soda, but has not taken them as often as directed. I ordered them to be continued as often as necessary to relieve pain.

January 12th—Bowels have moved, probably from the effects of the powders, though not all of the four grains of calomel have yet been taken; pains more general; complains of shoulders; skin moist; tongue coated; white; moist; urine sufficient in quantity, and normal in quality; pulse 96, or a little below 100 per minute. Made no change in the treatment.

January 13th—The condition about the same; rheumatic difficulty more decidedly migratory; the powders of calomel, morphine and soda prescribed on the 10th have been renewed—without my direction, however; does not take much food; has all the time been worse during the night than during the day; no change in treatment.

January 14th—I had been quite sick for two or three days, and on the 14th was unable to leave my bed. I heard nothing from the case till the morning of the 15th, when I learned from Dr. F. H. Davis and Dr. Lester Curtis that they had both seen her on the evening of the 14th. Dr. Curtis made the following prescription:

℞.—Potass. bromid., ʒ ii.

Aquæ,

Syr. cort. aurant } aa fl. ℥ i.

M. S. One teaspoonful every hour, as directed.

I am told that she took one dose of the mixture prescribed by Dr. Curtis, after which Dr. Davis was sent for. He ordered the following:

℞.—Potass. bromid., ʒ iv.

Cryst. carbolic acid, grs. vi.

Glycerine, fl. ℥ ss.

Aquæ, fl. ℥ iiiss.

M. One teaspoonful every three hours.

This was continued during the night, and till I saw her on the 15th. The powders were continued, as they were judged necessary to allay pain.

I saw her about 2 P. M. They said that Dr. Davis's medicine had given her great relief. Some muscular tenderness yet over the chest and in the shoulders; pulse about 100; regular, and moderately full. Directed the continuance of the bromide of potassa and carbolic acid solution. Saw her again in the evening.

January 16th—Less positive pain, but had a restless night—only "snatches of sleep," as she expressed it. Has not taken the powders since yesterday. Directed the following:

℞.—Tr. opii camph., fl. ℥ i.

S. Half teaspoonful every two hours, if awake. Continue the bromide and carbolic acid solution. She only took one dose of the paregoric.

In the evening she begged for something to relieve the pain, which had become much more decided, especially in the limbs and joints; could hardly move in bed. I gave her a hypodermic injection of morphia, $\frac{1}{4}$ grain, and atropin $\frac{1}{60}$ grain; she went to sleep in about five minutes, and slept quietly until three or four o'clock in the morning, after which she was restless; slept between seven and eight hours.

January 17th—Found her in the morning absolutely helpless; arms, hands, fingers, hips, knees, ankles, and toes even, tender, and most of the joints somewhat swollen; skin hot, but still moist; tongue moist; urine scanty; pulse over 100, hard, small, but regular; heart sounds normal in quality. Directed the following:

℞.—Tinct. colchici., fl. ʒ i.

Potass. bi carb., ʒ ii.

Potass. cit., ʒ iii.

Aquæ camph., fl. ℥ ii.

Syr. limon, fl. ℥ iv.

M. ft. sol. Take one tablespoonful every two hours.

This prescription was continued during the day. She was a little flighty when left alone, but recovered perfectly when spoken to, and answered intelligently questions addressed to her. In the evening, about the same; has not slept during the day; in fact, not much since three or four A. M.; gave again a hypodermic injection of morphine, $\frac{1}{5}$ grain, and atropin, $\frac{1}{75}$ grain.

January 18th, A. M.—Limbs less painful and tender; can move them a little; pulse about 95; skin moist, and cooler than last night; slept between six and seven hours, under the influence of the injection of morphine and atropin; I directed the colchicum mixture to be continued.

Afternoon — Bowels have moved freely during the day; copious watery discharges; limbs better—can use them quite well; complains of pain, mostly in right shoulder; is very weary, and wants sleep; discontinued colchicum mixture, and gave, between three and four o'clock, a hypodermic injection of morphine, $\frac{1}{5}$ grain, and atropin $\frac{1}{75}$ grain.

January 19th, A. M.—Found her free from pain in joints; had slept several hours during the night, but was still restless, anxious; talking a little incoherently, but answering when spoken to; pulse about 100; urine scanty; micturition difficult and painful. Directed the following:

℞.—Potass. acet., ʒ iij.

Potass. bromid., ʒ ij.

Spts. nit. dulc., fl. ℥ i.

Syr. limon, fl. ℥ v.

M. ft. sol. Take one tablespoonful every two hours until relieved.

Afternoon—Has passed urine freely, and without pain; skin quite moist, but hot; pulse more frequent—115 per minute, regular; suspected cardiac trouble; found heart-sounds normal in quality; no articular pain, except in right shoulder, and this not severe. Discontinued diuretic, and ordered the following:

℞.—Sat. rad. aconit., fl. ʒ i.

Morph. sulph., grs. ij.

Syr. simp., fl. ℥ ij.

M. ft. sol. Take one teaspoonful every two hours.

Saw her three times during this day. She slept during the afternoon and evening only a few minutes at a time, and then would start up suddenly, saying something irrelevant, but, upon being spoken to, becoming perfectly conscious.

January 20, morning. Pulse 90, soft, moderately full, regular in rythm. Mr. S. says that after my first visit last night the pulse went up to 120 per minute. Skin moist, not so hot as during the day of the 19th; urine free in quantity; tongue moist; little or no pain anywhere; stomach tolerates milk.

10 P. M. Pulse 84, regular, soft, moderately full. Skin moist and cool, but not cold; apparently normal in its qualities. Has had no pain during the day. During the last twenty hours has had no continuous quiet sleep. Has been taking during the day half-teaspoonful doses of the aconite and morphine mixture. For the purpose of procuring sleep, I directed the following:

℞.—Chloral hydrate, ʒ i.

Syr. simp. fl. ℥ ii.

Glycerine, fl. ℥ i.

Chloroform, fl. ʒ ss.

M. ft. sol.

S. Dose, one tablespoonful.

Of this mixture one tablespoonful was ordered to be given every hour till she slept. She took but one dose, and soon afterwards became wildly delirious. At 10 P. M., or about that time, I saw her again. The pulse was then 104, and somewhat irregular, small and weak; breathing oppressed; panting; vomited fluid matters, apparently milk and mucous, immediately after I came in. Bowels also moved; did not see the stool. She was helped out of bed; did not seem to have any pain; in answer to questions, complained only of nausea and oppression of epigastrum. She had been now twenty-four hours without continuous sleep, and I gave, for the purpose of producing it, a hypodermic injection of morphine gr. $\frac{1}{5}$ and atropine gr. $\frac{1}{75}$. She went to sleep in about eight minutes. The pulse became more regular, at 102 per minute; breathing easier, but not free and full. The temperature of the skin seemed to be normal, and was neither dry nor excessively moist.

At 3 A. M. of the 21st I was sent for, and upon arriving I found her dead. Mr. S. said that she had slept continuously from the time I left; that between 2 and 3 A. M. they turned her in bed, and that she suddenly ceased to breathe.

She had nourishment as the stomach would tolerate it. During the last day or two, milk was grateful; and she took as much of it as she liked.

Throughout her sickness she was worse at night.

No. 4 Sixteenth street, Chicago.

*February*, 1873.

---

REMARKS BY THE SENIOR EDITOR.

The foregoing simple history of the symptoms and treatment of a case, the fatal termination of which caused the husband of Mrs. S. to recently issue a card in a daily paper, charging the death to the "recklessness and carelessness" of the attending physician, was placed in our hands for publication before such card appeared.

The case is of intrinsic interest to the profession, as it evidently represents a class, not numerous, yet extremely liable to deceive the physician and disappoint the friends. From the foregoing history it appears that the patient presented all the ordinary symptoms of subacute articular and muscular rheumatism, in a highly nervous temperament, with no indications of either cardiac or cerebral complications until the 19th of January.

On the morning of that day the joints are represented as free from pain or soreness, except one shoulder; and yet the pulse was 100 per minute, and increased during the day to 115, with some incoherence, and such a degree of nervous restlessness as to justify the suspicion that the rheumatic irritation was developing in the muscular structure of the heart — although no valvular murmurs were audible.

The active administration of aconite in combination with morphine, apparently checked the cardiac excitement, and brought the pulse to 90 on the following morning, and to 84 at 6 o'clock in the evening, with entire freedom from pain and fever. But, the patient being still wakeful and restless, the attending physician thought it important to procure for her a night of sleep, and for that purpose ordered about *ten grains* of hydrate of chloral in solution with five drops of chloroform, to be given every hour until sleep was induced; while aconite was to be temporarily omitted. Soon after the first dose of the chloral was given, the patient began to be delirious; and, instead of repeating the dose every hour until she became quiet, as ordered, the nurse gave nothing more, allowing the excitement to increase rapidly until 10 o'clock, P. M., when the physician was sent for. By the time he arrived the delirium was excessive, the breathing oppressed, and the pulse rapid, very irregular and feeble. Feeling certain that a continuance of the excitement and agitation of the patient would end in fatal exhaustion in a few hours, the attending physician resorted immediately to the hypodermic injection of morphia and atropia, as the most speedy and certain mode of restoring quiet. He was the more ready to do this, as the same means had been resorted to with good effects during the patient's illness. The dose or quantity used was only a medium one, and under its effects the patient not only slept, but the pulse became more regular and the respiration more natural; and we have no doubt but that she lived longer than she would have done if the wild excitement had been allowed to continue. That the sudden supervention of delirium, accompanied by hurried or oppressed breathing, with a small, rapid and irregular pulse, fully justified the opinion of the physician that her death was caused by rheumatic carditis, we have no doubt.

If our readers will take the trouble to refer to the last edition of "Watson's Practice," Vol. II., pages 235–6, 338–40–41, 759 and 766, they will find full corroboration of this view, and citation of closely parallel cases. Further corroboration may be found in the first English edition of "Aitken's Practice," Vol. II., pages 573 and 579.

We do not quote from these authors, because the works are in the hands of most of our readers.

Jaccoud, one of the most celebrated professors in the medical faculty at Paris, in his work on practical medicine ("*Pathologie Interne*"), says of the complications of acute articular rheumatism: "The most important are *inflammation of the heart and its envelopes*. . . . Rheumatic endocarditis may, like all other forms of the disease, produce embolia, infiltiatious and intracardiac coagulations. These results of the cardiac complications are the principal causes of rapid or sudden death sometimes observed in the course of rheumatism." In reference to the cerebral symptoms, the same author says: "Sometimes during the existence of acute articular rheumatism, cerebral accidents are suddenly observed, which are, for the most part, rapidly mortal." For some of these cases, the author accounts upon the hypothesis of embolia due to cardiac complications—for others, by the presence of meningeal punctiform hæmorrhages. "But," he says, "in a certain number of cases the autopsy is mute; it shows no sufficient lesion of the encephalon, or of its investments. The clinical picture is always the same in its fundamental features. The patient is taken with restlessness, with active delirium of variable violence, falls into coma, and dies in a few hours, or after a day or two. Sometimes the delirium is absent, or only transient, and the coma is the initial fact; death is then still more rapid."—*Jaccoud Pathologie Interne, Tome II., page* 547 *and* 549, *of Paris*, 1872.

Raynaud, in the "*Norveau Dictionniare de Med. and Chirurg. Pratique*," article "*Cœur*," says of inflammation of the substance of the heart, that "It is often observed secondarily in the course of an acute or chronic articular rheumatism; and that it is consecutive to endocarditis, or pericarditis" (page 469, Vol. VIII). "The symptoms are often very uncertain" (page 470, Vol. VIII). "Endocarditis is not always revealed by physical signs" (page 365, same volume). "The affections of the heart occur much more rarely in rheumatism affecting a single joint than in cases where a large number of joints are simultaneously attacked" (page 366, same volume).

Niemeyer, French Edition, Vol. II., page 582, says of the termination of acute rheumatism: "Sometimes death occurs without complications, and with the indications of sudden collapses, having been preceded for a short time by delirium, or coma, and other symptoms of a grave disturbance of innervation."

Very many other authorities might be cited, all confirmative of the statements of Watson, as to the fact of sudden death occurring in the course of acute rheumatism, preceded for a short time by delirium or coma. His opinion that in such cases death is usually produced by the cardiac trouble is, I think, also sustained.

The authorities here cited are of the highest standing; and, instead of "carelessness" or "recklessness" on the part of the attending physician, as charged by Mr. S., the history of the case shows a commendable degree of faithfulness, careful attention, and skill in the selection and application of remedies.

---

Emotional Insanity.—At a recent meeting of the New York Medico-Legal Society, David Dudley Field, Esq., in discoursing upon "Emotional Insanity," concluded as follows:

Children under the age of discretion, idiots and imbeciles are not within the discipline of criminal law.

The mental unsoundness of other persons, commonly designated as insanity or mania, is in itself, or is attended by, disease of the brain, so that no heat of mere passion, and no degree of mere frenzy, can in any just sense be pronounced insanity by either of the professions.

That neither perceptional nor emotional insanity by itself, nor both together, can be accepted as excuse for criminal responsibility.

That intellectual or volitional insanity absolves from criminal responsibility when, and only when, the reason has lost either the power of choice or the power of controlling the will.

That in every case of acquittal on the ground of insanity, the defendant should be forthwith placed in a lunatic asylum, and there kept until it is proved that he is restored to such a state of sanity as to remove all apprehension of a recurrence of the disease.

That the present gradation of punishments is unsuited to the present condition of medical learning, and a change is required which shall make the law punish, not only according to the harmfulness of the outward act, but according to the quality of the inward spring of action.—*Medical Record.*

Depilatory Mixture.—A new depilatory mixture is recommended by Prof. Boettger, consisting of one part of crystalized sulphydrate of sodium, and three parts of prepared chalk. Mixed with water, and applied to the skin, it effects the easy removal of hair.—*Med. and Surg. Reporter.*

